# Transposable elements in Rosaceae: insights into genome evolution, expression dynamics, and syntenic gene regulation

**DOI:** 10.1093/hr/uhae118

**Published:** 2024-04-26

**Authors:** Ze Yu, Jiale Li, Hanyu Wang, Boya Ping, Xinchu Li, Zhiguang Liu, Bocheng Guo, Qiaoming Yu, Yangjun Zou, Yaqiang Sun, Fengwang Ma, Tao Zhao

**Affiliations:** State Key Laboratory for Crop Stress Resistance and High-Efficiency Production/Shaanxi Key Laboratory of Apple, College of Horticulture, Northwest A&F University, Yangling, Shaanxi 712100, China; State Key Laboratory for Crop Stress Resistance and High-Efficiency Production/Shaanxi Key Laboratory of Apple, College of Horticulture, Northwest A&F University, Yangling, Shaanxi 712100, China; State Key Laboratory for Crop Stress Resistance and High-Efficiency Production/Shaanxi Key Laboratory of Apple, College of Horticulture, Northwest A&F University, Yangling, Shaanxi 712100, China; State Key Laboratory for Crop Stress Resistance and High-Efficiency Production/Shaanxi Key Laboratory of Apple, College of Horticulture, Northwest A&F University, Yangling, Shaanxi 712100, China; State Key Laboratory for Crop Stress Resistance and High-Efficiency Production/Shaanxi Key Laboratory of Apple, College of Horticulture, Northwest A&F University, Yangling, Shaanxi 712100, China; State Key Laboratory for Crop Stress Resistance and High-Efficiency Production/Shaanxi Key Laboratory of Apple, College of Horticulture, Northwest A&F University, Yangling, Shaanxi 712100, China; State Key Laboratory for Crop Stress Resistance and High-Efficiency Production/Shaanxi Key Laboratory of Apple, College of Horticulture, Northwest A&F University, Yangling, Shaanxi 712100, China; State Key Laboratory for Crop Stress Resistance and High-Efficiency Production/Shaanxi Key Laboratory of Apple, College of Horticulture, Northwest A&F University, Yangling, Shaanxi 712100, China; State Key Laboratory for Crop Stress Resistance and High-Efficiency Production/Shaanxi Key Laboratory of Apple, College of Horticulture, Northwest A&F University, Yangling, Shaanxi 712100, China; State Key Laboratory for Crop Stress Resistance and High-Efficiency Production/Shaanxi Key Laboratory of Apple, College of Horticulture, Northwest A&F University, Yangling, Shaanxi 712100, China; State Key Laboratory for Crop Stress Resistance and High-Efficiency Production/Shaanxi Key Laboratory of Apple, College of Horticulture, Northwest A&F University, Yangling, Shaanxi 712100, China; State Key Laboratory for Crop Stress Resistance and High-Efficiency Production/Shaanxi Key Laboratory of Apple, College of Horticulture, Northwest A&F University, Yangling, Shaanxi 712100, China

## Abstract

Transposable elements (TEs) exert significant influence on plant genomic structure and gene expression. Here, we explored TE-related aspects across 14 Rosaceae genomes, investigating genomic distribution, transposition activity, expression patterns, and nearby differentially expressed genes (DEGs). Analyses unveiled distinct long terminal repeat retrotransposon (LTR–RT) evolutionary patterns, reflecting varied genome size changes among nine species over the past million years. In the past 2.5 million years, *Rubus idaeus* showed a transposition rate twice as fast as *Fragaria vesca*, while *Pyrus bretschneideri* displayed significantly faster transposition compared with *Crataegus pinnatifida*. Genes adjacent to recent TE insertions were linked to adversity resistance, while those near previous insertions were functionally enriched in morphogenesis, enzyme activity, and metabolic processes. Expression analysis revealed diverse responses of LTR–RTs to internal or external conditions. Furthermore, we identified 3695 pairs of syntenic DEGs proximal to TEs in *Malus domestica* cv. ‘Gala’ and *M. domestica* (GDDH13), suggesting TE insertions may contribute to varietal trait differences in these apple varieties. Our study across representative Rosaceae species underscores the pivotal role of TEs in plant genome evolution within this diverse family. It elucidates how these elements regulate syntenic DEGs on a genome-wide scale, offering insights into Rosaceae-specific genomic evolution.

## Introduction

Transposable elements (TEs) represent mobile DNA sequences pervasive across the genomes of most eukaryotes [1]. Their role in shaping plant genomes is notably diverse and impactful. Successive cycles of expansion and contraction in TE quantity serve as catalysts for significant disparities in the overall genomic architecture, even among closely related plant species [2]. TEs constitute a predominant, and often the predominant, portion of the entire plant genome [3, 4]. Moreover, TE activity contributes to a wide spectrum of change in gene expression and functionality. This ranges from subtle quantitative influences to substantial diversification of gene regulatory networks (GRNs) and even the emergence of entirely novel genes [5–8].

TEs within plant genomes exhibit a diverse array of structures and configurations [9]. The prevalent classification system categorizes TEs based on transposition mechanisms and enzymological criteria, broadly dividing them into two principal classes: Class I (retrotransposons, RTs) and Class II (DNA transposons) [10]. Retrotransposons, within Class I, employ a ‘copy-and-paste’ transposition mechanism involving RNA intermediates, while DNA transposons, under Class II, utilize a ‘cut-and-paste’ method through DNA intermediates [11]. Class I elements, transcribed by RNA polymerase II (RNA Pol II), generate mRNA that is converted into cDNA by reverse transcriptase (RT). This cDNA is then integrated at a new locus by an integrase (INT) [12], facilitating genome expansions. Generally, Class I elements are comprised of five orders, named long terminal repeat retrotransposons (LTR–RTs), *DIRS*-like elements, Penelope-like elements, LINEs (long interspersed nuclear elements), and SINEs (short interspersed nuclear elements) [10]. Depending on the distinct mechanisms of integration, Class I elements can be divided into LTR retrotransposons and non-LTR retrotransposons [1, 13]. Among the numerous superfamilies of LTR retrotransposons, the most common are the *Copia* (RLC) and *Gypsy* (RLG) superfamilies. However, some novel LTR retrotransposons belong to the ‘Unknown’ superfamily (RLU) due to the deficiency of a coding sequence [14]. Moreover, the families of LTR retrotransposons (LTR–RTs) have been suggested as a taxonomic category encompassing shared structural and functional characteristics, as well as evolutionary connections [15]. Each family of LTR–RTs is a clade of members that possess high DNA sequence similarity (>80%) in their internal regions encoding proteins related to transposition [10]. There are numerous families of LTR–RTs found within plant genomes, e.g. *Ale*, *Alesia*, *Angela*, *Bianca*, *Ikeros*, *Ivana*, *SIRE*, *TAR*, *Tork*, *Athila*, *CRM*, *Galadriel*, *Ogre*, *Reina*, *Retand*, and *Tekay* [16, 17]. According to whether the retrotransposons can transpose independently, retrotransposons fall into two types: autonomous retrotransposons and non-autonomous retrotransposons. The latter include LINEs and SINEs, which are more common within animal genomes. Class II elements have four orders, comprising TIR (terminal inverted repeats), Crypton, Helitron, as well as Maverick. TIR elements and MITEs (miniature inverted repeat transposable elements, defined as non-autonomous versions of TIR elements) have five familiar superfamilies, including *hAT* (DTA), *CACTA* (DTC), *PIF-Harbinger* (DTH), *Mutator* (DTM), and *Tc1-Mariner* (DTT) [10]. Additionally, because of their special transposition mechanism, *Helitron* superfamily (DHH) members have been widely studied by scientists [10].

TEs can act as regulatory units, reshaping gene expression upon insertion into specific loci, thereby potentially inducing phenotypic variations. For instance, in *Arabidopsis thaliana* experiencing proteotoxic stress, genes proximal to SINEs alter their expression patterns, thereby rewiring stress-related gene regulatory networks [8]. The impact of TE insertions on phenotypic traits is evident in various plant varieties. In ‘Chardonnay’ grapes, an original insertion of a *Gret1* LTR retrotransposon led to a loss-of-function allele of the *Vvmyb1A* gene, resulting in green fruit. Subsequent rearrangements in *Gret1* converted green fruit to red fruit in varieties like ‘Ruby Okuyama’ [18]. Similarly, the insertion of a *Gypsy*-like LTR–RT named redTE upstream of the *MdMYB1* gene in apples distinguished red fruit color in HFTH1 from yellow fruit color of GDDH13 [19]. Recently discovered LTR–RTs, such as HODOR (high-copy ‘Golden Delicious’ repeat), have garnered significant scientific interest due to their association with high DNA methylation levels [20]. Additionally, a methylated MITE insertion (MITE-MdRF1) in the promoter of *MdRFNR1-1*, when exposed to drought stress, is recognized by transcriptional anti-silencing factors, thereby promoting *MdRFNR1-1* expression [21]. Conversely, in a white-fruited *Fragaria vesca* wild type, an insertion of a *Gypsy* LTR–RT into *FvMYB10* truncated the production of *FvMYB10*, impeding the anthocyanin biosynthesis pathway [22]. However, the activity of TEs is predominately subdued through epigenetic modification such as DNA methylation, small RNA interference, and histone modification, crucial for sustaining genome integrity [23, 24]. TEs can be rejuvenated under biotic and abiotic stress by the derepression of silent epigenetic conditions or through the action of transcription factors [25, 26]. This reactivation of TEs often leads to phenotypic plasticity and aids in defense against detrimental natural selection by regulating the expression of genes surrounding their insertion sites.

The Rosaceae family boasts a cosmopolitan distribution; it is characterized by a diverse morphology and holds substantial economic and ecological significance. Known for its wide range of genome sizes [27], this family encompasses numerous renowned species of both financial and scientific importance, including apples, pears, hawthorns, loquats, raspberries, *Gillenia trifoliata*, peaches, strawberries, *Dryas drummondii*, and a variety of ornamental flowers like roses, meadowsweets, and hawthorns [28]. This diverse array of species within Rosaceae provides an exceptional resource for comparative analysis. The availability of genome and transcriptome datasets for Rosaceae [29, 30] has facilitated extensive investigations into the genome evolution of this family on a genome-wide scale. Notably, the abundance of TEs in Rosaceae genomes varies significantly. For instance, TE sequences represent ~22% of the genome in strawberry (*F. vesca*) [31], 29.60% in peach (*Prunus persica*) [32], 47.20% in *G. trifoliata* [33], 57.30% in apple (*Malus domestica* GDDH13) [20], and 66.03% in hawthorn (*Crataegus pinnatifida* var. *major*) [34]. This substantial variation in TE content across Rosaceae genomes underscores the dynamic nature of TE proliferation and their potential impact on the genomic architecture of these diverse plant species.

In this study, we aim to conduct a comprehensive identification and thorough characterization of TEs across 12 Rosaceae species. We systematically compared the genomic composition, insertion patterns, and functional impact on nearby genes attributed to LTR–RTs across the Rosaceae species. This comparative approach allowed us to elucidate the co-evolutionary relationships between LTR–RTs and their host genomes. Furthermore, we conducted an in-depth analysis of the transcriptional activity of LTR–RTs to discern the specific genes influenced by highly expressed LTR–RTs. Additionally, we performed differential gene expression analysis to pinpoint candidate TEs that might significantly impact these differentially expressed genes (DEGs). Our comprehensive approach aimed to unravel the intricate interplay between LTR–RTs and the genomic landscape of Rosaceae species, shedding light on their potential regulatory roles in gene expression and evolutionary dynamics within this diverse plant family.

## Results

### Transposable element diversity within Rosaceae

**Figure 1:**
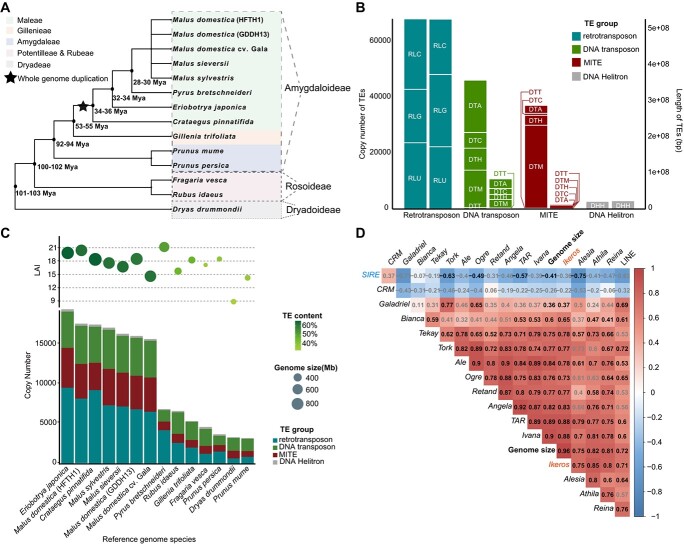
TE distribution across 14 representative Rosaceae genomes. **A** The 14 Rosaceae genomes used in this study (phylogeny adapted from Xiang *et al*. [28]). **B** Copy numbers (left axis) and total length (right axis) of TEs across all 14 genomes. The smaller boxes within the bars represent TE superfamilies, each identified by a three-letter code based on a common TE classification system [10]. The segments are categorized into Retrotransposons, DNA transposons, MITEs, and DNA *Helitron* transposons. The labels RLC, RLG, RLU, DTA, DTC, DTH, DTM, DTT, and DHH correspond to *Copia* LTR–RT, *Gypsy* LTR–RT, Unknown LTR–RT, *hAT* DNA transposon, *CACTA* DNA transposon, *PIF–Harbinger* DNA transposon, *Mutator* DNA transposon, *Tc1–Mariner* DNA transposon, and *Helitron* DNA transposon, respectively. **C** LTR assembly index (LAI), genome size, TE content, and TE copy number for each species. Circle sizes reflect genome sizes, while degrees of shading indicate the TE content. **D** Spearman correlation matrix showing the relationship between 16 LTR–RT families and genome size. Shades of darkness represent the strength of the correlation, with stronger positive or negative correlations represented by darker levels of shading. Bold gray font represents *P* ≥ 0.05, while regular black font denotes *P* < 0.05.

Our study analyzed 14 representative and available genomes in Rosaceae (Fig. 1), encompassing 12 species; note that we used three cultivars for *M. domestica* (Fig. 1). These tested species exhibit varying speciation times, spanning from 28 to 103 million years ago (MYA) [28]. Notably, the Maleae lineage’s origin can be traced back to a whole-genome duplication (WGD) event in an ancestor closely related to *Gillenia* (*x* = 9) [32, 35–37]. The genome sizes of the tested Rosaceae species examined in this study showcase remarkable diversity: e.g. ~825 Mb for *C. pinnatifida* (17 chromosomes) [34], ~760 Mb for *Eriobotrya japonica* (17 chromosomes) [38], ~660 Mb for *M. domestica* (HFTH1) (17 chromosomes) [19], ~510 Mb for *Pyrus bretschneideri* (17 chromosomes) [39], ~295 Mb for *Rubus idaeus* (7 chromosomes) [40], ~280 Mb for *G. trifoliata* (9 chromosomes) [38], ~235 Mb for *D. drummondii* [41], ~230 Mb for *P. persica* (8 chromosomes) [32], and ~220 Mb for *F. vesca* (7 chromosomes) [42].

Intact TEs contain complete structural features. For an intact LTR element, this includes the left target site duplication, left LTR, internal region producing reverse transcriptase, right LTR, and right target site duplication. For an intact TIR element, it contains the left target site duplication, left terminal inverted repeat, internal region producing several transposase enzymes, right terminal inverted repeat, and right target site duplication [42]. We classified intact TEs into distinct categories: *Copia* LTR–RT/*Gypsy* LTR–RT/Unknown LTR–RT, *hAT* DNA transposon/*CACTA* DNA transposon /*PIF–Harbinger* DNA transposon/*Mutator* DNA transposon/*Tc1–Mariner* DNA transposon, MITE, and *Helitron* DNA transposon (Fig. 1B). Among the analyzed genomes, *E. japonica* and *D. drummondii* exhibited the highest (65.15%) and lowest (33.47%) TE content, respectively. Notably, *E. japonica* possessed the second largest genome size (761.57 Mb), while *D. drummondii* had the third smallest genome size (232.91 Mb) (Fig. 1C). To shed light on the impact of intact TE numbers on genome expansion, we performed a correlation analysis between various TE hierarchies and genome size across 12 Rosaceae species. Our analysis revealed that the number of retrotransposons displayed the strongest positive correlation with genome size at the order level (Spearman’s *r* = 0.98, *P* < 0.01) (Supplementary Data Fig. S1). At the family level, the quantity of *Ikeros Copia* LTR–RTs demonstrated the most robust positive correlation with genome size (Spearman’s *r* = 0.96, *P* < 0.05). Conversely, the abundance of *SIRE Copia* LTR–RTs exhibited a relatively negative correlation with genome size (Spearman’s *r* = −0.41, *P* < 0.05) (Fig. 1D).

### Distinct activity of LTR–RTs in the nine Rosaceae genomes

**Figure 2:**
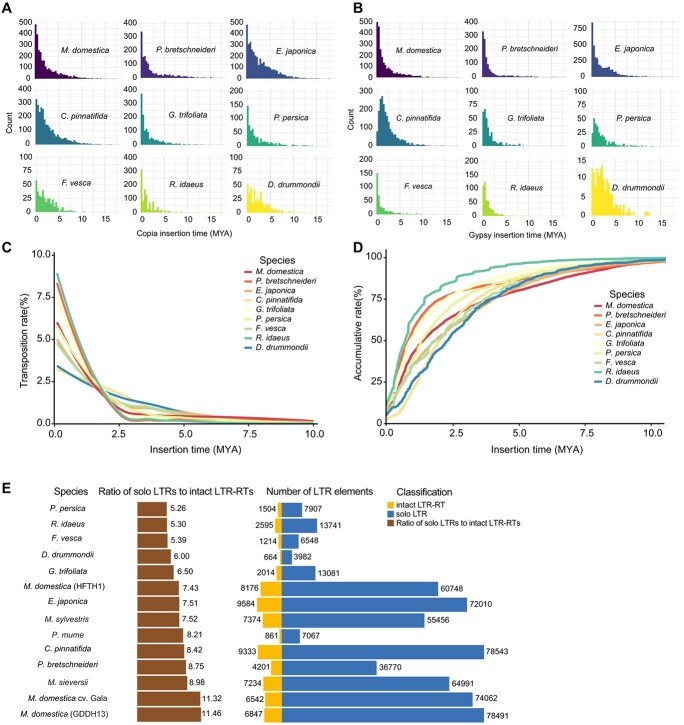
Distinct activity of LTR–RTs across Rosaceae genomes. **A** Distribution of insertion times for *Copia* LTR–RTs within nine species. **B** Distribution of insertion times for *Gypsy* LTR–RTs across the same nine species. **C** Transposition rates of LTR–RTs in the nine species. This rate is defined as the ratio of the net increase in LTR–RTs observed every 0.1 MY relative to the total LTR–RTs over a 10-MY time-scale. **D** Accumulation rates of LTR–RTs in increments of 0.1 MY over a 10-MY period. *M. domestica* (HFTH1) was used to represent *M. domestica* in panels **A**–**D**. **E** Comparison of solo LTRs and intact LTR–RTs of LTR elements across the 14 Rosaceae genomes.

We conducted estimations of TE insertion times, revealing distinctive activity patterns among *Copia* and *Gypsy* LTR–RTs within Maleae, Gillenleae, Rubeae, Potentilleae, and Dryadeae in Rosaceae genomes. Notably, the estimated burst, indicated by median age, of *Copia* LTR–RTs occurred later than that of *Gypsy* LTR–RTs in Maleae, Gillenleae, and Rubeae. In contrast, the burst of *Copia* LTR–RTs preceded that of *Gypsy* LTR–RTs in Potentilleae and Dryadeae (Fig. 2A and B). Moreover, the calculated insertion times of *Copia* LTR–RTs are predominantly concentrated at 0 MYA, indicating recent insertions, which are similar to most of those *Gypsy* LTR–RTs, except for *C. pinnatifida*, *G. trifoliata*, *P. persica*, *R. idaeus*, and *D. drummondii*. For these species, the insertion times were concentrated on 1.3, 0.7, 0.7, 0.7, and 2.5 MYA, respectively.

The interplay between DNA removal and TE proliferation constitutes a dynamic process that influences the evolution of genome size, leading to either expansion or shrinkage. To compare TE activity, we calculated the transposition rate, representing the net increase in the total number of LTR–RTs within every 0.1 million years (MY) over a 10-MY period (Fig. 2C). Over the past 2.5 MY, the transposition rates of *R. idaeus*, *P. bretschneideri*, and *G. trifoliata* have notably surged, while those of *M. domestica*, *P. persica*, *E. japonica*, and *F. vesca* have moderately increased. Conversely, the rates in *D. drummondii* and *C. pinnatifida* have remained relatively stable. A similar trend was observed in the cumulative rate of LTR–RTs within every 0.1 MY over a 10-MY scale across the nine genomes (Fig. 2D).

DNA removal has been hypothesized to play a dominant role in hindering TE proliferation-mediated genome expansion [43, 44]. Intact LTR–RTs with a pair of identical direct repeats are specifically favored for DNA removal via unequal homologous recombination (HR) events because the two LTRs provide homologous regions to initiate illegitimate recombination [45, 46]. Frequent HR-mediated DNA removal may lead to a high abundance of solo LTR remnants in the genome, which can serve as evidence supporting the existence of an inherently efficient DNA removal mechanism. Therefore, we compared the ratio of solo LTRs to intact LTR–RTs among the 14 Rosaceae genomes (Fig. 2E). The respective abundances of solo LTR and intact LTR–RTs were used to evaluate the propensity of HR-mediated removal of active LTR insertions in the 14 genomes. The ratios of solo LTRs to intact LTR–RTs in *P. persica*, *R. idaeus*, *F. vesca*, *D. drummondii*, and *G. trifoliata* are relatively low, ranging from 5.26 to 6.5. The ratios in *M. domestica* (HFTH1), *E. japonica*, *Malus sylvestris*, *Prunus mume*, *C. pinnatifida*, *P. bretschneideri*, and *Malus sieversii* are comparatively moderate, ranging from 7.43 to 8.98. The ratios in *M. domestica* (GDDH13), and *M. domestica* cv. ‘Gala’ are considerably high, ranging from 11.32 to 11.46. It is worth noting that, in Maleae, the ratios of solo LTRs to intact LTR–RTs in *M. domestica* (GDDH13), *M. domestica* cv. ‘Gala’, *M. sieversii*, and *P. bretschneideri* are higher than those in *C. pinnatifida* and *E. japonica*. This suggests that *M. domestica* (GDDH13), *M. domestica* cv. ‘Gala’, *M. sieversii*, and *P. bretschneideri* might possess a highly efficient, inherent molecular mechanism to purge LTR–RTs, probably through HR-mediated DNA removal, thus accelerating the processes of genome size shrinking. This result is in line with the fact that genome sizes of *C. pinnatifida* and *E. japonica* are much larger than those of *M. domestica* (GDDH13), *M. domestica* cv. ‘Gala’, *M. sieversii*, and *P. bretschneideri*.

### Functions of TE-proximal genes in nine Rosaceae species

**Figure 3:**
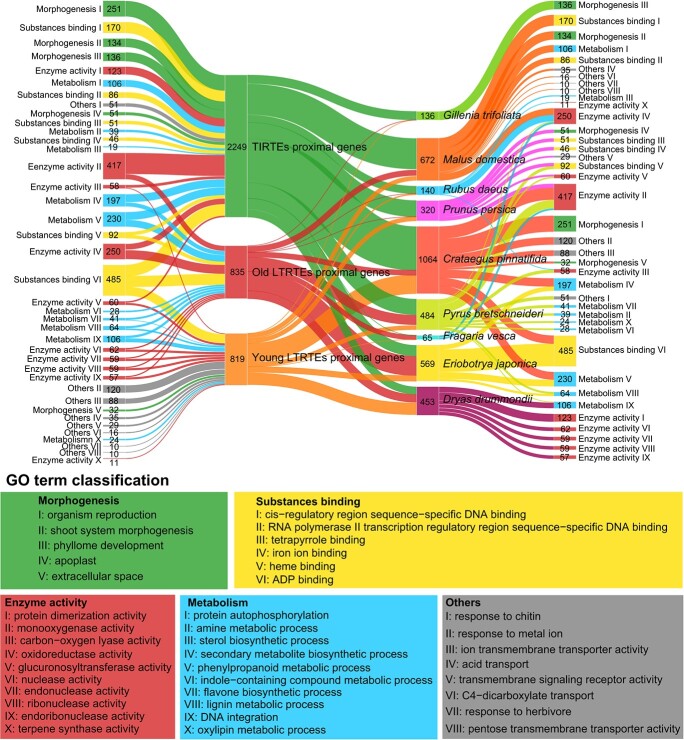
Sankey diagram illustrating the inferred functions of TE-proximal genes. The width of connections between each vertical block represents the gene count, delineating GO term classifications associated with TE-proximal genes, the number of genes adjacent to three distinct types of TEs, genes categorized across nine species, and the GO term classifications of TE-proximal genes. TE-proximal gene GO terms are categorized into five primary groups: morphogenesis, substance binding, enzyme activity, metabolism, and others. Each group encompasses various detailed GO term descriptions representing specific gene functions.

Gene expression patterns are intricately regulated by enhancers and repressors, whether they are located nearby or at a distance. Movement of genes mediated by TEs to new chromosomal contexts harbors the potential to alter gene regulation and reshape the genome architecture [1]. To delve into the potential functions of TE-proximal genes, we conducted Gene Ontology (GO) enrichment analysis for genes adjacent to specific intact TEs. Our analysis revealed distinct functional categories associated with these genes, which can be classified into five primary types: plant morphogenesis, substance binding, influence on enzyme activity, metabolism and synthesis, and functional attributes (Fig. 3). This categorization highlights the diverse roles played by TE-proximal genes, shedding light on their potential impact on various biological processes within the genome.

The functions attributed to TIRTE-proximal genes encompass a diverse range, including effects on enzyme activity (27.97%), substance binding (25.74%), plant morphogenesis (25.43%), and metabolism and synthesis (18.59%), with a smaller fraction falling under other functional categories (2.27%). In contrast, the characteristics associated with genes proximal to young LTR–RTs (representing the top 50% of the total insertion time of LTR–RTs) exhibit varying proportions, comprising others (37.61%), enzyme activity (25.52%), substance binding (17.46%), and metabolism and synthesis (15.51%), and a smaller percentage associated with plant morphogenesis (3.91%). Conversely, genes proximal to old LTR–RTs (representing the latter 50% of the total insertion time of LTR–RTs) predominantly display attributes related to enzyme activity (38.09%) and metabolism and synthesis (37.00%), with a smaller fraction associated with substances binding (24.91%) (Fig. 3).

In *G. trifoliata*, genes adjacent to a TE exhibit an enrichment in the GO term associated with plant morphogenesis (Fig. 3). Conversely, in *R. idaeus* and *F. vesca*, the enriched GO term is primarily linked to enzyme activity. *Eriobotrya japonica* showcases genes related to metabolism and synthesis, along with substance binding, while in *D. drummondii* genes are associated with metabolism and synthesis, as well as enzyme activity (Fig. 3). Notably, *M. domestica*, *P. bretschneideri*, *C. pinnatifida*, and *P. persica* exhibit genes linked to four or more distinct functional types, highlighting a broader functional diversity among the genes proximal to TEs within these species (Fig. 3).

### Spatiotemporally specific LTR–RT expression in *M. domestica* cv. ‘Gala’

TE activity has the potential to significantly impact gene family evolution, particularly through the actions of retrotransposons. Retrotransposons have been identified as contributors to gene family expansion by transporting neighboring genes and integrating them into different genomic locations during their transposition [47]. In our investigation into the activity of LTR–RTs, we initially classified all intact LTR–RTs into two distinct groups: ‘domain-existent’ and ‘structure-intact’. This classification was based on the integrity of coding regions associated with five essential domains: capsid protein (GAG), aspartic proteinase (AP), integrase (INT), reverse transcriptase (RT), and RNAse H (RH). Domain-existent LRT–RTs are presumed to possess at least one of these five domains, while structure-intact LTR–RTs are likely to contain all five domains. Specifically focusing on *M. domestica* cv. ‘Gala’, we identified 222 specifically expressed domain-existent LTR–RTs and 100 specifically expressed structure-intact LTR–RTs, shedding light on the distinct expression patterns and potential activity of these retrotransposon groups within this particular apple cultivar.

**Figure 4:**
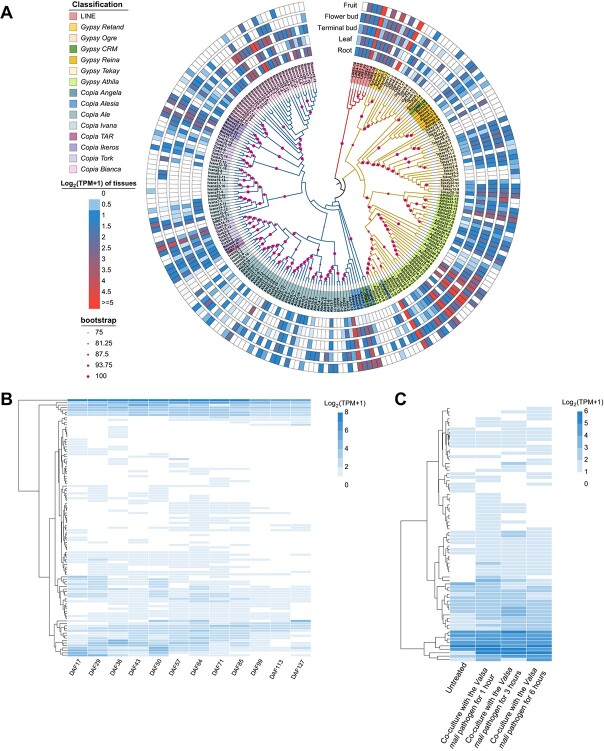
Spatiotemporally specific expression patterns of 222 domain-existent retrotransposons within the genome of *M. domestica*. **A** Highly expressed retrotransposons in *M. domestica* genome. The heat maps represent the log_2_-transformed transcripts per million (TPM + 1) values of retrotransposons across different tissues, including fruit, flower bud, terminal bud, leaf, and root. The phylogenetic tree classified the transposons into different clades. **B** Expression patterns of these retrotransposons at various developmental stages of apple fruit. **C** Expression profiles of these domain-existent retrotransposons during exposure to *Valsa mali *pathogen for 1, 3, and 6 hours, applied to apple leaves.

We further classified the domain-existent LTR–RTs into distinct clades based on sequence similarities using the maximum likelihood (ML) method. Through this analysis, we identified a total of eight *Copia* clades (*Ale*, *Alesia*, *Angela*, *Bianca*, *Ikeros*, *Ivana*, *TAR*, and *Tork*), six *Gypsy* clades (*Athila*, *CRM*, *Ogre*, *Reina*, *Retand*, and *Tekay*), and one LINE clade within all LTR–RT sequences in *M. domestica* cv. ‘Gala’ (Fig. 4A). These clades exhibited considerable variation in size, the number of elements ranging from 1 to 38 for the *Copia* clades and from 2 to 42 for the *Gypsy* clades. This categorization based on sequence similarities offers insights into the diversity and distribution of distinct clades within the LTR–RT sequences identified in this specific apple cultivar.

The expression profiles of transposed members within these clades across different tissues provide a basis for estimating the transposition activity of LTR–RTs (Fig. 4A). In *M. domestica* cv. ‘Gala’, when analyzing various tissues, including fruit, flower bud, terminal bud, leaf, and root, we observed that certain LTR–RT expressions were tissue-specific. For instance, Ogre2-1, Athila32-15, and Bianca30-sc, among others, exhibited tissue-specific expression patterns. Conversely, other LTR–RTs, like LINE2-7, and Retand3-11, showed no tissue-specific expression. Furthermore, we visualized differentially expressed LTR–RTs at specific time points after full bloom (17, 29, 36, 43, 50, 57, 64, 71, 85, 99, 113, and 127 days) in the fruit of *M. domestica* cv. ‘Gala’. Certain LTR–RTs displayed temporal specificity, such as Athila40-sc and Tekay10-7. However, some LTR–RTs, such as Ogre2-1 and Athila15-10, exhibited consistently high expression levels without temporal specificity (Fig. 4B, Supplementary Data S4). Moreover, our investigation delved into the potential impact of adversity stress on LTR–RT expression differences. Analyzing leaf cells of *M. domestica* cv. ‘Gala’ after co-culture with the *Valsa mali* pathogen for varying durations (1, 3, and 6 h), we observed differential expression of specific LTR–RTs, including Athila32-15 and Ale12-7, under the stress conditions. Conversely, other LTR–RTs, like Alesia3-5 and LINE4-13, showed no differential expression under the same conditions (Fig. 4C, Supplementary Data S4). Similar trends were noticed in the expression patterns of structure-intact LTR–RTs, mirroring those of domain-existent LTR–RTs (Supplementary Data Fig. S2). These findings highlight the potential influence of tissue types, developmental stages, and environmental stresses on the expression dynamics of distinct LTR–RTs within the genome of *M. domestica* cv. ‘Gala’.

### Distinct gene expression in *M. domestica* cultivars ‘Gala’ and ‘Golden Delicious’ driven by cultivar-specific transposable elements

**Figure 5:**
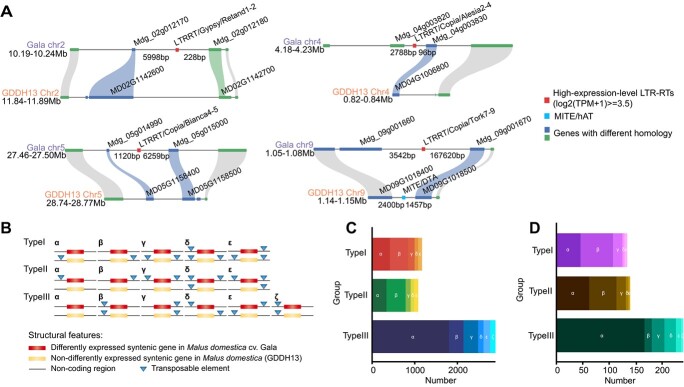
TE distribution near genes in *M. domestica*. **A** Micro-synteny visualization exhibiting high-expression-level transcripts of domain-existent LTR–RTs in *M. domestica* cv. ‘Gala’ and *M. domestica* (GDDH13). Highly-expressed-LTR–RTs (log_2_(TPM + 1) ≥ 3.5) were shown. **B** Classification scheme illustrating the positional and quantitative relationship between TEs and DEGs. **C** Count of syntenic and period-specific genes in *M. domestica* cv. ‘Gala’ and *M. domestica* (GDDH13) (log_2_(fold change) ≥ 1.5 of two adjacent periods in *M. domestica* cv. ‘Gala’ fruit on days 29, 36, 43, 57, 71, 85, 99, 113, and 127; log_2_(fold change) < 1.5 of two adjacent periods in *M. domestica* (GDDH13) fruit on days 28, 35, 42, 56, 70, 84, 98, 112, and 126). **D** Count of syntenic and period-specific genes in *M. domestica* cv. ‘Gala’ and *M. domestica* (GDDH13) (TPM > 32 in *M. domestica* cv. ‘Gala’ fruit on days 29, 36, 43, 57, 71, 85, 99, 113, and 127; TPM < 8 in *M. domestica* (GDDH13) fruit on days 28, 35, 42, 56, 70, 84, 98, 112, and 126).

The insertion of TEs within genomic regions can lead to diverse impacts on gene expression. These elements might diminish gene expression by interrupting the normal structure of a gene [48]. Conversely, they can also potentially elevate gene expression, given that TEs encompass various *cis*-regulatory elements capable of providing novel regulatory modules that activate gene expression [49]. Here, we identified four domain-existent LTR–RTs with notably high expression levels {log_2_ [transcripts per million (TPM) + 1] ≥ 3.5} positioned adjacent to genes, where the distance between the LTR–RTs and the genes was <5000 bp in *M. domestica* cv. ‘Gala’ (Fig. 5A). However, in the syntenic region of chromosome 9 of *M. domestica* (GDDH13) we did not observe any TEs but instead found a MITE (Fig. 5A).

To explore the association between TEs and DEGs, we conducted a synteny analysis involving ‘Gala’ and GDDH13. Initially, we constructed synteny detection using all genes in ‘Gala’ and GDDH13. Subsequently, we identified sets of period-specific highly expressed genes in ‘Gala’ fruit [log_2_(fold change) ≥ 1.5 of two adjacent periods] and period-specific non-highly expressed genes in GDDH13 fruit [log_2_(fold change) < 1.5 of two adjacent periods] from specific days. We then cross-referenced this information with the synteny data and counted the number of TEs in proximity to the DEGs in both cultivars. We categorized the positional relationships between TEs (not considering inside introns or exons) and genes into three main types and 16 subtypes (Fig. 5B). Type I and Type II classifications are based on the discrepancy in the number of TEs near a DEG between the two cultivars, while Type III signifies the absence of such differences. Among the 5157 pairs of time-specific DEG pairs, 1173 pairs belonged to Type I, 1083 pairs belonged to Type II, and 2901 pairs were classified under Type III (including 1811 pairs categorized as Type IIIα) (Fig. 5C). Additionally, we identified cultivar-specific highly expressed genes in ‘Gala’ fruit and cultivar-specific lowly expressed genes in GDDH13 fruit. A total of 517 pairs of cultivar-specific DEG pairs were identified, comprising 135 pairs of Type I, 140 pairs of Type II, and 242 pairs of Type III (including 168 pairs classified as Type IIIα) (Fig. 5D). For a detailed view of the syntenic DEGs and their proximal genetic elements, please refer to the provided list (Supplementary Data S3).

## Discussion

Comparative genomics has emerged as a powerful approach to unravel diverse evolutionary events shaping plant evolution, including alterations in gene expression, chromosomal rearrangements, and gene transposition. As the sequencing of plant genomes continues, our understanding of genome evolution across different species in the plant kingdom has expanded significantly. Besides the ongoing polyploidization of plant species, the activity of TEs stands as a crucial driver contributing to the vast diversity observed in plant genome sizes, especially among closely related species sharing a similar evolutionary history [47]. The coding regions among closely related plant species exhibit similarity; however, the distribution and proportions of TEs, notably retrotransposable elements residing in intergenic regions, display substantial diversity. Our exhaustive TE analyses, comparing 12 Rosaceae species across metrics such as number distribution, insertion age, transposition preferences, functional domains, phylogenetic categorization, and expression profiling, highlight the expression of LTR–RTs in response to environmental shifts. Furthermore, our findings suggest that differential gene expression may be a consequence of TE insertions.

However, it is important to recognize the present limitations in sequencing and TE annotation technologies, which might result in incomplete identification of TEs in genomes enriched with repetitive sequences. Hence, our analysis primarily centered on intact retrotransposons bearing identifiable paired LTRs or distinctive domains, alongside transposons displaying clear structural characteristics. This focused approach aimed to mitigate potential errors arising from assembly inaccuracies. As updated versions of reference genomes for Rosaceae organisms become available in the future, conducting further analyses will offer an opportunity to validate the conclusions drawn from this study.

Recent studies focusing on the *Arabidopsis*, *Eutrema*, *Oryza*, and *Helianthus* genera have similarly highlighted the strong correlation between retrotransposable element activity and the evolution of genome size [47, 50, 51]. These investigations indicate that estimated insertion times of LTR–RTs can serve as indicators of evolutionary trends, elucidating whether plant genomes have expanded or shrunk [50, 51]. Considering the rates of transposition, accumulation of LTR–RTs and the ratio of solo LTRs to intact LTR–RTs in the nine Rosaceae genomes (Fig. 2), and assuming that the rate of DNA loss countering TE insertions in *D. drummondii* and *C. pinnatifida* reflects the general pace of genome evolution in the Rosaceae family, it is highly plausible that the absence of LTR–RT accumulation in *F. vesca* over the last 2 MY suggests a significant decline in TE activity, thereby facilitating genome shrinkage. In contrast, the *P. bretschneideri* genome displays fewer LTR–RTs with young insertion ages, indicating a recent surge in LTR–RT proliferation and pronounced DNA loss within the past 2 MY, resulting in rapid genome shrinkage. Our findings illustrate that by scrutinizing recent LTR–RT activity and the ratio of solo LTRs to intact LTR–RTs we can infer patterns in genome size evolution within a relatively short evolutionary timeframe in plants.

In this study, we focused on 12 closely related, fully sequenced species within the Rosaceae family to explore the relationship between genome structure and TEs. Initially, we anticipated finding a similar number of intact LTR–RTs in *P. bretschneideri*, *M. domestica*, *C. pinnatifida*, and *E. japonica* due to their shared evolutionary trajectories. While determining the precise direction of species evolution throughout an extensive history is challenging, inferring the evolutionary trend based on the latest transposition events of specific TE classes is feasible [47]. Surprisingly, our observations did not align with this expectation. This disparity suggests the possibility that *P. bretschneideri* and other Maleae plants might have adopted distinct molecular mechanisms to manage extensive TE transposition despite sharing a common evolutionary history: *P. bretschneideri* appears to have employed an active mechanism, swiftly removing deleterious LTR–RT insertions through preferential DNA removal, whereas, *M. domestica*, *C. pinnatifida*, and *E. japonica* seem to have employed a passive mechanism, confining harmless and outdated *Gypsy* insertions to gene-poor heterochromatin regions. These differing TE defense strategies have led to the development of large genomes (such as those observed in *M. domestica*, *C. pinnatifida*, *E. japonica*, and *R. idaeus*), characterized by extensive centromere expansion, and smaller genomes (notably in *P. bretschneideri* and *F. vesca*) undergoing rapid genome downsizing.

TEs have been recognized to influence the expression of various genes, significantly impacting plant evolution [49, 52]. In perennial fruit species, TE insertions adjacent to genes have been found to affect numerous agronomic traits, including parthenocarpic apple fruit [5], increased fruit size in apples [53], red-skinned phenotype of apples [19], blood orange formation [54], somatic embryogenesis in citrus [55], obstruction of fruit development in grapevine [56], generation of somatic variations in grapevine cluster shape [57], and response to drought stress in apple [21]. In this study, we identified and selected 3903 genes adjacent to intact TEs and examined their potential functions using GO enrichment analysis (Fig. 3). Given the ‘cut-and-paste’ transposing mechanism of DNA transposons, all DNA transposons are likely to be aged. Genes neighboring aged TEs are predominantly associated with plant morphogenesis, enzyme activity, and metabolic processes, whereas genes proximal to young TEs are focused on resistance to adversity and substance transport across membranes. Over the course of evolution, species may eliminate aged TEs that compromise adaptability to the environment through DNA removal or purifying selection, while maintaining ancient TEs that play a foundational role for adaptations, such as transcription factor binding sites (TFBSs) and enhancer-like elements in genes. Conserved TE sequences persisting at specific sites for extended periods are often repurposed and warrant further investigation. Under harsh environmental conditions, adversity stress is likely to trigger TE activation, as most TEs remain silent in a genome through epigenetic silencing [23, 24]. Consequently, a recently inserted TE near specific genes might play a pivotal role in adapting to challenging environmental factors.

Typical intact autonomous LTR–RTs involve several essential proteins for transposition. While our analysis delves into the variations of LTR–RT expression across different tissues, developmental stages of fruit, and various experimental treatments (Fig. 4), the underlying mechanism governing the modulation of LTR–RT expression under distinct conditions warrants further investigation. Additionally, the actual expression of these LTR–RTs in *M. domestica* cv. ‘Gala’ requires experimental verification. Future validation may involve the adoption of new measurement methods for assessing LTR–RT expression or the utilization of updated versions of RNA-sequencing (RNA-seq) data. Reanalysis using these advancements will be necessary to validate and reinforce the conclusions drawn in the present study.

The insertion of TEs within and around genes has been known to lead to allele-specific expression (ASE) [48]. Building on this concept, we conducted a meticulous analysis of context elements associated with syntenic DEGs in two dimensions: varying developmental stages and distinct varieties. Our investigation categorized the positional relationships between TEs and syntenic DEGs into the 16 types previously mentioned based on the number of TEs neighboring the genes. We observed and classified 5157 pairs of syntenic DEGs in *M. domestica* cv. ‘Gala’ and *M. domestica* (GDDH13) across different stages of fruit development, and similarly categorized 517 pairs of syntenic DEGs in these varieties. In total, 3695 pairs of syntenic and TE-proximal DEGs were identified in this study, significantly contributing to future genomic research and molecular breeding in apples. The innovative method devised in this study for rapid quantification of LTR–RTs holds promise for application in other plant species with high-quality genomes. Its utilization is poised to accelerate our comprehension of the role of TEs in plant evolution, crop domestication, and enhancement.

### Conclusions

This study presents a comprehensive investigation into the genomic evolution of 14 representative Rosaceae plants facilitated by TEs. Specifically, the distinct evolutionary dynamics of LTR–RTs reflect the different patterns of genome size changes in Rosaceae species over the past million years. Genes adjacent to recent TE insertions are associated with adversity resistance, while those near previous insertions are functionally enriched in morphogenesis, enzyme activity, and metabolic processes. Expression analysis reveals diverse responses of LTR–RTs to internal or external conditions. Additionally, 3695 pairs of syntenic DEGs proximal to TEs in *M. domestica* cv. ‘Gala’ and *M. domestica* (GDDH13) suggest that TE insertions may contribute to varietal trait differences in these apple varieties. These findings shed light on the pivotal role of TEs in plant genome evolution within the diverse Rosaceae family.

## Materials and methods

### Identification of TEs in 14 genomes of Rosaceae species

All reference genomes were downloaded from public repositories, including GDR, NCBI, and CNGB (Supplementary Data S1). The Extensive *de novo* TE Annotator (EDTA), LTR_retriever, and TEsorter were used to annotate and classify whole-genome intact TEs and solo LTRs [16, 58–67]. The LTR assembly index (LAI) and the insertion time of LTR–RTs were calculated by LTR_retriever [64, 68]. These calculations were based on the Rosaceae mutation rate, approximated at ~4 × 10^−9^ mutations per site per year [69].

### Gene Ontology enrichment of TE-proximal genes

GO enrichment analysis was performed using the R package clusterProfiler (v4.2.2) [70], *q*valueCutoff was selected as 0.2. As mentioned above, TEs were divided into young LTR–RTs, old LTR–RTs, and TIR TEs. We conducted a GO enrichment analysis on genes proximal to three types of TE across the nine species. The top five GO terms, determined by the largest number of associated genes, were utilized as input data for generating the Sankey plot. The Sankey plot visualization was created using the R package sankeyD3 (v0.3.2).

### Quantification of TE expression

The apple genome (*M. domestica* cv. ‘Gala’) harbors a diverse array of Class I order retrotransposons, and benefits from ample transcriptome data available for analysis. To explore expression variations within Class I retrotransposon families, we focused on the apple genome as a model system. We gathered 21 distinct expression datasets from NCBI, encompassing various tissues, developmental stages of fruit, and responses to pathogens. These datasets were consolidated, resulting in a comprehensive transcriptome dataset of 61 samples (Supplementary Data S2). Utilizing pseudoalignment methods applied to RNA-seq data by Kallisto (version 0.48.0) [71], we quantified the expressions of structurally intact TEs across diverse conditions. The coding regions of structurally intact TEs were retrieved using the gff2seq.py script from TEsorter [58]. Both coding regions of TEs and genome-wide annotated genes are used as the reference required in Kallisto.

### Multiple sequence alignment and phylogenetic analysis

The GAG, AP, INT, RT, and RH domains of identified LTR–RTs were used for phylogenetic analysis. Sequence alignment analysis was performed using MAFFT (v7.310) with default parameters [72]. ML trees were constructed for the trimmed alignments with IQ-TREE (v.2.0.3) using ModelFinder for the best-fitting evolutionary model and UFBoot2 for branch support values [73–75]. The resulting phylogenetic trees were visualized with iTOL [76]. To unravel the evolutionary trajectory of retrotransposons in *M. domestica*, we curated a dataset comprising 222 domain-existent retrotransposon (including 100 structure-intact retrotransposon) sequences exhibiting a minimum TPM value >1 across the various conditions mentioned above. This dataset was utilized to reconstruct a novel phylogenetic relationship within *M. domestica* cv. ‘Gala’.

### Synteny analysis of genes

The synteny relationship of genes in ‘Gala’ and GDDH13 was generated with the SynNet-Pipeline, which is available at https://github.com/zhaotao1987/SynNet-Pipeline [77]. To visualize highly expressed LTR–RTs and their proximal genes, we used JCVI to achieve microsynteny visualization [78].

### Statistical analyses

Correlation statistics were calculated using the stats package in R. We used the R package rstatix (v0.7.2) for Spearman testing of the number of TEs and genome sizes.

## Supplementary Material

Web_Material_uhae118
