# Supplementary material for: Transposable elements in Rosaceae: insights into genome evolution, expression dynamics, and syntenic gene regulation
Source: Hortic Res. 2024 Apr 26;11(6):uhae118. doi: 10.1093/hr/uhae118 (PMC11197308; doi:10.1093/hr/uhae118)
Supplement: Web_Material_uhae118 [file web_material_uhae118.zip › FigS2.pdf]

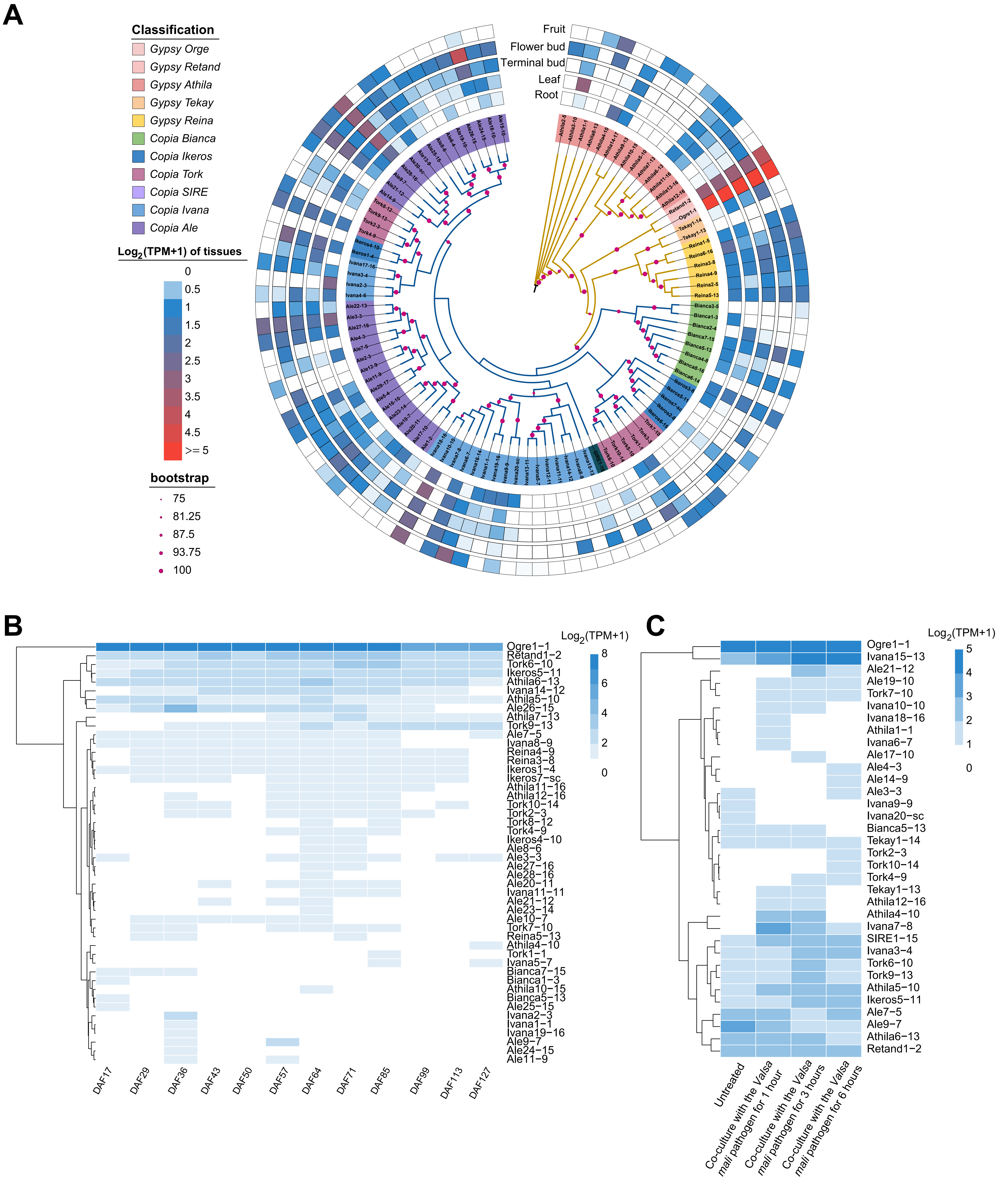
A
Classification
Gypsy Orge
Gypsy Retand
Gypsy Athila
Gypsy Tekay
Gypsy Reina
Copia Bianca
Copia Ikeros
Copia Tork
Copia SIRE
Copia Ivana
Copia Ale
Log2(TPM+1) of tissues
0
0.5
1
1.5
2
2.5
3
3.5
4
4.5
>= 5
bootstrap
75
81.25
87.5
93.75
100
Fruit
Flower bud
Terminal bud
Leaf
Root
B
Log2(TPM+1)
8
6
4
2
0
Ogre1−1
Retand1−2
Tork6−10
Ikeros5−11
Athila6−13
Ivana14−12
Athila5−10
Ale26−15
Athila7−13
Tork9−13
Ale7−5
Ivana8−9
Reina4−9
Reina3−8
Ikeros1−4
Ikeros7−sc
Athila11−16
Athila12−16
Tork10−14
Tork2−3
Tork8−12
Tork4−9
Ikeros4−10
Ale8−6
Ale3−3
Ale27−16
Ale28−16
Ale20−11
Ivana11−11
Ale21−12
Ale23−14
Ale10−7
Tork7−10
Reina5−13
Athila4−10
Tork1−1
Ivana5−7
Bianca7−15
Bianca1−3
Athila10−15
Bianca5−13
Ale25−15
Ivana2−3
Ivana1−1
Ivana19−16
Ale9−7
Ale24−15
Ale11−9
DAF17
DAF29
DAF36
DAF43
DAF50
DAF57
DAF64
DAF71
DAF85
DAF99
DAF113
DAF127
C
Log2(TPM+1)
5
4
3
2
1
0
Ogre1−1
Ivana15−13
Ale21−12
Ale19−10
Tork7−10
Ivana10−10
Ivana18−16
Athila1−1
Ivana6−7
Ale17−10
Ale4−3
Ale14−9
Ale3−3
Ivana9−9
Ivana20−sc
Bianca5−13
Tekay1−14
Tork2−3
Tork10−14
Tork4−9
Tekay1−13
Athila12−16
Athila4−10
Ivana7−8
SIRE1−15
Ivana3−4
Tork6−10
Tork9−13
Athila5−10
Ikeros5−11
Ale7−5
Ale9−7
Athila6−13
Retand1−2
Untreated
Co-culture with the Valsa mali pathogen for 1 hour
Co-culture with the Valsa mali pathogen for 3 hours
Co-culture with the Valsa mali pathogen for 6 hours
