# Supplementary figures and images for: Transposable elements in Rosaceae: insights into genome evolution, expression dynamics, and syntenic gene regulation
Source: Hortic Res. 2024 Apr 26;11(6):uhae118. doi: 10.1093/hr/uhae118 (PMC11197308; doi:10.1093/hr/uhae118)

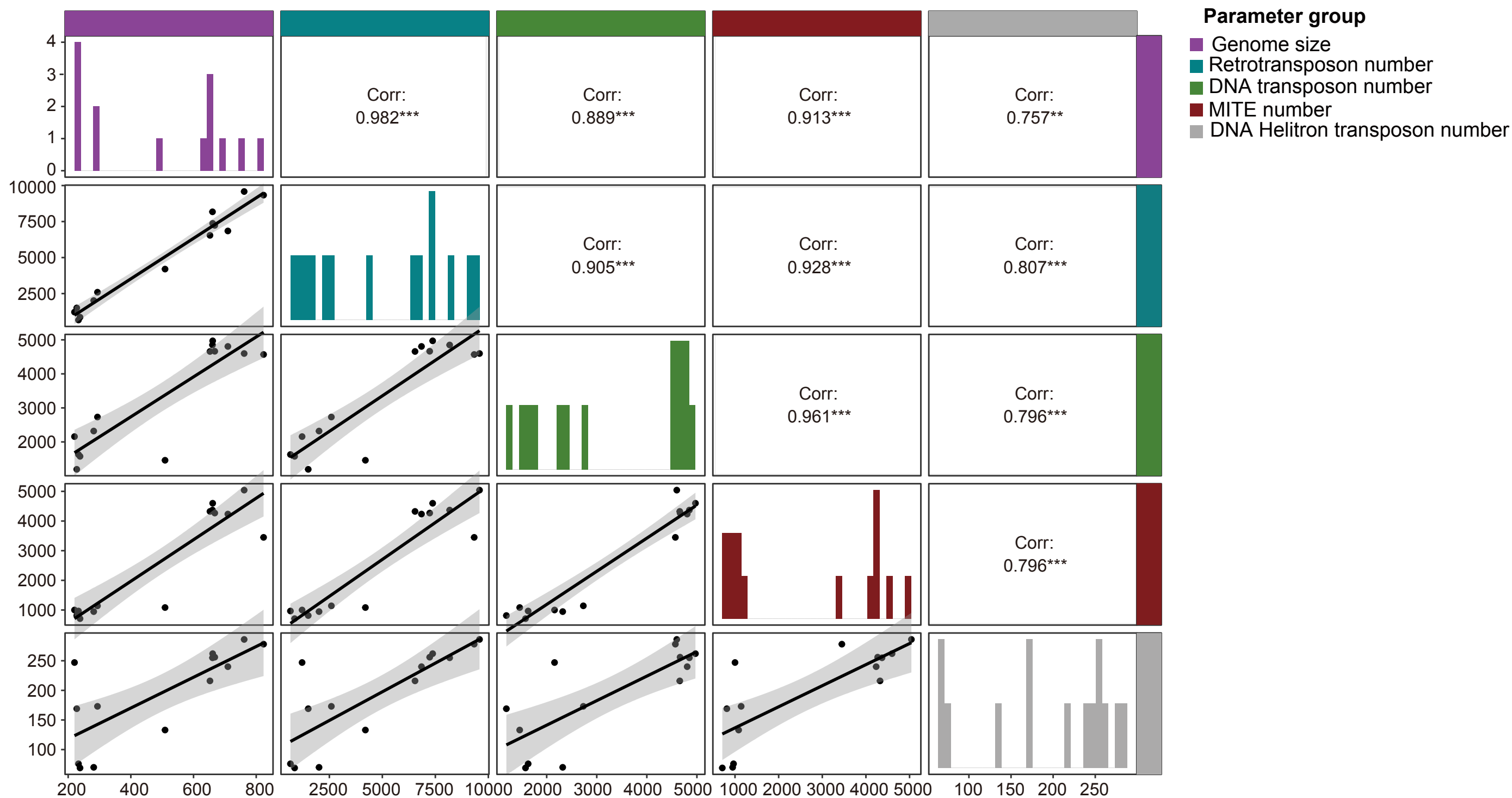

Supplement: Web_Material_uhae118 [file web_material_uhae118.zip › FigS1.pdf]
